# Theranostic 3-Dimensional nano brain-implant for prolonged and localized treatment of recurrent glioma

**DOI:** 10.1038/srep43271

**Published:** 2017-03-06

**Authors:** Ranjith Ramachandran, Vijayabhaskar Reddy Junnuthula, G. Siddaramana Gowd, Anusha Ashokan, John Thomas, Reshmi Peethambaran, Anoop Thomas, Ayalur Kodakara Kochugovindan Unni, Dilip Panikar, Shantikumar V. Nair, Manzoor Koyakutty

**Affiliations:** 1 Amrita Centre for Nanosciences and Molecular Medicine, Amrita University, Kochi, 682041, Kerala, India; 2 Central Lab Animal Facility, Amrita Institute of Medical Sciences & Research Centre, Amrita University, Kochi, 682041, Kerala, India; 3 Department of Neurosurgery, Amrita Institute of Medical Sciences & Research Centre, Amrita University, Kochi, 682041, Kerala, India

## Abstract

Localized and controlled delivery of chemotherapeutics directly in brain-tumor for prolonged periods may radically improve the prognosis of recurrent glioblastoma. Here, we report a unique method of nanofiber by fiber controlled delivery of anti-cancer drug, Temozolomide, in orthotopic brain-tumor for one month using flexible polymeric nano-implant. A library of drug loaded (20 wt%) electrospun nanofiber of PLGA-PLA-PCL blends with distinct *in vivo* brain-release kinetics (hours to months) were numerically selected and a single nano-implant was formed by co-electrospinning of nano-fiber such that different set of fibres releases the drug for a specific periods from days to months by fiber-by-fiber switching. Orthotopic rat glioma implanted wafers showed constant drug release (116.6 μg/day) with negligible leakage into the peripheral blood (<100 ng) rendering ~1000 fold differential drug dosage in tumor versus peripheral blood. Most importantly, implant with one month release profile resulted in long-term (>4 month) survival of 85.7% animals whereas 07 day releasing implant showed tumor recurrence in 54.6% animals, rendering a median survival of only 74 days. In effect, we show that highly controlled drug delivery is possible for prolonged periods in orthotopic brain-tumor using combinatorial nanofibre libraries of bulk-eroding polymers, thereby controlling glioma recurrence.

Despite many new drugs having been developed and tested for high-grade glioma (Glioblastoma Multiforme or GBM), the overall survival of patients remains dismal at 12–15 months with a three year long term survival of 3–5%^1^,^2^,^3^. There are many reasons for the poor outcome with new drugs: (i) limited ability of drugs to cross the blood brain barrier (BBB)^4^,^5^, in spite of the compromised BBB in glioma patients, (ii) poor drug accumulation in the brain tumor site^6^ (iii) inability to sustain adequate drug concentration in the tumor due to early dissipation into the cerebrospinal fluid (CSF) and interstitial fluid (ISF)^7^, and (iv) short half-life of drug, limits the diffusion distance from the polymer implant^8^. In addition, other factors such as the presence of glioma stem cells^9^ and drug resistance due to genomic mutations such as EGFR vIII too contribute to glioma recurrence^10^. In more than 90% of clinical cases, the tumor recurrence happens within 2 cm region of the resected margin^11^,^12^,^13^,^14^. This suggests that localized and sustained delivery of chemo drugs directly into the tumor bed for prolonged periods without leakage into the peripheral blood, would be an ideal scenario for treating glioblastoma^15^,^16^,^17^.

Direct drug delivery into the brain using polymeric implants^18^,^19^,^20^, microparticles^21^, microcapsules^22^, microchips^23^ and nanoparticles^24^,^25^ has been an important topic of GBM research because of their potential for sustained drug release. However, a specific post-surgical solution was the clinically successful implantable wafer, Gliadel^™^, which is placed into the tumor-resected cavity. It is the only FDA-approved intracranial drug delivery system available in the clinic for the last two decades^26^,^27^,^28^,^29^,^30^. Pioneering work done by Henry Brem and Robert Langer *et al*., lead to the development of this pelletized microparticle implant, formed by a surface-eroding polyanhydride, *p*-Carboxyphenoxy propane-sebacic acid (CPP-SA), which delivers oncolytic agent, Carmustine (BCNU), for nearly 7 days (90%). This provided an improved median survival advantage of ~2.3 months^31^. Although, Gliadel remains to be the model system for intracranial drug delivery, tumor recurrence has been reported in majority of treated cases, predominantly, at the site of resected primary tumor. This suggests that 7-day release may be inadequate to counter recurrence. In addition, the limited tissue penetration of Carmustine (1–5 mm) and its short half-life (20 min.) are major limiting factors for sustained therapeutic effects. Clearly, there exists an urgent necessity to improve the prolonged drug delivery for anti-glioma drugs^32^,^33^.

At the research level, many polymeric systems loaded with Temozolomide^34^,^35^,^36^, Paclitaxel^19^,^37^,^38^, Doxorubicin^39^,^40^, 5-fluorouracil^41^ and Camptothecin^42^, were reported in experimental models, however, prolonged delivery, especially within the intracranial tumor micro-environment, for more than 2–3 weeks remains a critical challenge. Notably, Tseng *et al*. has reported the use of electrospun PLGA nanofibers for the delivery of Carmustine and other therapeutic agents (irinotecan, vancomycin and cisplatin) directly into the brain^43^,^44^,^45^,^46^,^47^. Although, drug release up to 8 weeks was reported, the site of implantation was not into the brain cortex. Instead, it was in the subarachnoid space (space between dura and pia matter that covers cerebral cortex). This region neither reflect the characteristics of actual brain tissue nor glioblastoma microenvironment, and resulted in the spread of drug throughout the brain up to contralateral region (probably by mixing with cerebral fluid), rendering no localization. In actual clinical scenario, drug-loaded implants are better placed in the tumor-resected cavity within the brain to manage the residual tumor cells. The tissue microenvironment at this region is completely different from that of subarachnoid regions, and hence, drug release profile may also vary significantly. Presence of residual tumor cells, inflammatory microenvironment, edema and necrotic fluid may accelerate biodegradation of polymer implants in the tumor cavity. In a recent report by Nance *et al*., convection-enhanced tissue penetrating PEGylated PLGA nanoparticles were reported for paclitaxel delivery in rat brain. However, this system had a burst release of 80% by 24 h and complete release by nearly 96 h^48^. Further, the maximum diffusion for nanoparticles by 24 h was nearly 400 μm. It is important that, compared to current daily oral dose of TMZ (150–200 mg/m^2^) providing 20–35% drug in brain^22^,^49^, local drug-delivery systems will have significance only if they are able to provide sustained release for at least a few weeks, for the same or higher dosage for prolonged periods without any systemic leakage. A major challenge in achieving this requirement, particularly in hostile tumor microenvironment, is the limited availability of surface-eroding polymers that are best suitable for controlled release. For the current clinical drug, Temozolomide (TMZ), the most promising result was reported by Brem *et al*., showing 60% drug release over 72 h and the rest by 144 h in rodent models, using the same surface-eroding polyanhydride polymer used in Gliadel^35^. Most of the other FDA-approved polymers such as PLGA, PLA, PCL and PVA are bulk-eroding polyesters, which exhibit either abrupt or negligible release owing to their characteristic degradation properties (too fast for PLGA or too slow for PLA/PCL). Thus, achieving a constant drug release within brain tumor for 1–2 months remains to be a challenging proposition.

Here, we report a simple and innovative method to overcome this challenge by creating a library of drug-loaded polyester nanofibers of PLGA-PLA-PCL blends and electrospun them together, to form a 3-dimensional (3D) composite nanofiber implant, capable of releasing anti-glioma drug Temozolomide (TMZ), continuously for one month into the brain tumor at a constant rate. The composition of each nanofibers in the implant was optimized using separate *in vivo* brain drug release experiments, and the data was used as ‘input’ to numerically design a composite implant formed from multiple nanofibers. The designed implant was experimentally realized through co-electrospinning of suitable polymer-drug blends into a common target, thus creating a 3D wafer, containing various nanofibers capable of releasing the drug for specific periods, ranging from one day to one month. The process of 3D spinning of pre-optimized polymer-drug blends as ‘ink’ is a novel, scalable and reproducible method for making custom-designed drug-eluting implants. We demonstrate two model implants: TMZ-FR for one week release and TMZ-SR for one month release. Our results clearly indicate that prolonged drug release in the brain tumor is critical in inhibiting the recurrence of glioblastoma. In addition, we also explain the potential of theranostic nanofibers for Magnetic Resonance Image (MRI) - guided *in vivo* implantation and non-invasive monitoring of the nano-device.

## Results and Discussion

### Preparation, characterization, *in vitro* and *in vivo* optimization of nanofiber library

**Figure 1:**
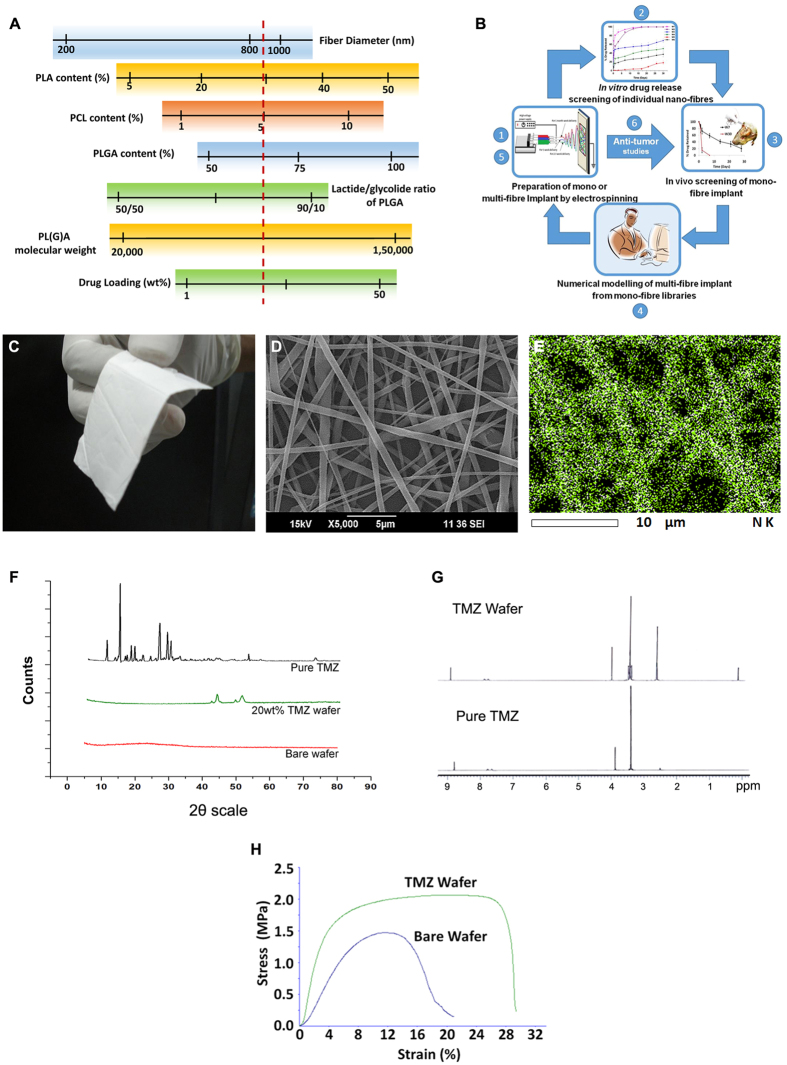
Preparation and physiochemical characterization of TMZ nanofiber implant. (**A**) Schematic diagram summarising the formulation parameters (polymer composition, molecular weight range, drug loading percentage and nanofiber diameter) of various wafer-implants prepared and evaluated during the optimization of TMZ loaded brain implants. TMZ-SR represents an optimized composition for one month release. (**B**) Schematic diagram showing overall developmental strategy used for developing nanofibrous implant for prolonged drug delivery in glioma. (**C**) Photograph and (**D**) SEM image of 20 wt% TMZ loaded nanofiber implant (W6) showing flexibility and smooth fiber morphology. (**E**) EDS mapping of drug showing uniform distribution of TMZ throughout the nanofibers. (**F**) XRD pattern of pure TMZ, bare wafer and 20 wt% TMZ loaded wafer showing encapsulation of TMZ in amorphous phase. (**G**) ^H^NMR spectrum of free and nanofiber loaded TMZ shows no modification in the chemical structure. (**H**) Stress-strain graph of bare and TMZ loaded implant shows enhanced flexible nature of the latter.

A library of nanofibers with characteristic *in vivo* drug release profile in the brain was prepared from three bulk eroding polymers, PLGA, PLA and PCL. Generally, bulk eroding polymers are not suitable for controlled drug release because of their abrupt biodegradation properties^50^. In order to overcome this, rationally selected polymer blends of both fast and slow-degrading polymers were used to optimize wide range of nanofiber compositions and generated a database of their *in vivo* release kinetics up to 2 months (Table S1, Supporting Information). TMZ loaded polymeric nanofibers were prepared with fiber diameters ranging from 200–1400 nm, molecular weights (20–150 kD), lactic to glycolic acid to caprolactone blend ratios (1:1:0 to 10.2:1:1.2) and percentage drug loadings (1–30 wt%) as depicted in Fig. 1A. The overall experimental plan is depicted in Fig. 1B. The nanofibers were prepared using electrospinning technique. TMZ release from individual compositions were studied, firstly *in vitro* using artificial cerebrospinal fluid (ACSF) and then *in vivo* using healthy and tumor bearing rat models. The *in vivo* data thus obtained was used as the ‘input’ to numerically design implants capable of releasing drug in constant rate for 7, 15 or 30 days. The designed implants were prepared by co-electrospinning and tested for brain release and anti-tumor efficacy in glioma bearing rats *in vivo*.

Figure 1C shows the representative photograph of TMZ loaded W6 nanofiber implant with lactide: glycolide: caprolactom ratio 6.7:1:0.5 and 20 wt% drug loading. SEM image (Fig. 1D) shows nanofibers with smooth surface morphology without any bead formation. Changes in the fiber diameter had great influence in the drug release properties, as better controlled release was possible with relatively higher fiber diameter than lower size-scale (Figure S1, Supporting Information). In another aspect, the burst release increased with percentage drug-loading. However, beyond 30 wt% drug loading, the fibers lost their morphology (Figure S2, Supporting Information). Nevertheless, irrespective of changes in the polymer composition, all wafers registered high encapsulation efficiency of 92.7–96.6% at 20 wt% drug loading. Energy dispersive X-ray (EDX) mapping (Fig. 1E) of TMZ showed uniform drug distribution in individual nanofibers. X-ray diffraction pattern (Fig. 1F), indicated that compared to crystalline phase of powder temozolomide, the fiber encapsulated drug was amorphous, signifying its better dissolution in polymer phase, critical for the controlled release^51^,^52^. The drug released from the implant is expected to dissolve in the interstitial fluid without re-crystallization and act against cancer cells. Exposure of drug molecule to various solvents and high voltage (30 kV) during electrospinning, may cause degradation of the drug during the implant preparation. NMR analysis of TMZ before and after electrospinning was carried out to examine the drug degradation (Fig. 1G). Both free and wafer loaded TMZ showed no changes in the chemical shifts peaks in ^1^H NMR spectrum ((DMSO-d6, δ): 8.80 (s, 1 H), 7.80 (bs, 1 H), 7.66 (bs, 1 H), 3.43 (s, 3 H)). Two additional peaks in NMR can be attributed to that of the polymer matrix. The flexibility of implant, without compromising its mechanical integrity, was another important aspect for easy application of implant in the brain cavity formed after tumor resection. Gliadel wafers were relatively rigid and brittle owing to the compression moulding process used for their preparation. In contrast, the electrospun wafers showed excellent flexibility, even with 20 wt% drug-loading, as shown in the tensile and flexural strength study (Fig. 1H). This improved flexibility will provide a great advantage for clinicians in implanting the wafer according to the macroscopic topography of the brain-cavity formed after tumor resection.

### *In vitro* release studies

**Figure 2:**
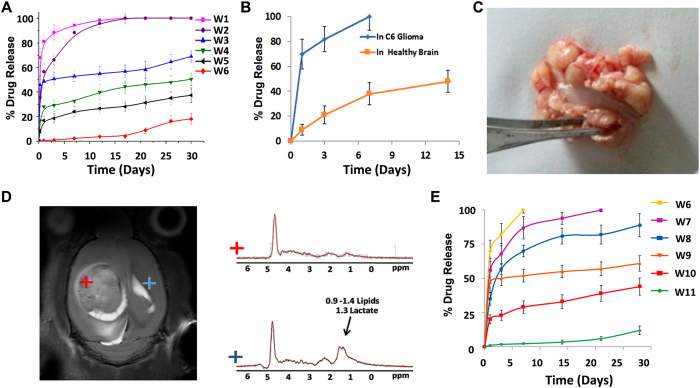
Nanofiber implant optimization using *in vitro* and *in vivo* drug release studies. (**A**) *In vitro* drug release profile of different polymer blends (W1 to W6) in artificial CSF. (**B**) Differential *in vivo* drug release behaviour of W6 implanted in normal brain versus rat C6 glioma. (**C**) Post-mortem analysis of tumor carried out one week after wafer implantation (W6, 20 wt% TMZ) showing no traceable implant within tumor due to aggressive degradation in tumor micro-environment. (**D**) NMR chemical shift imaging of intracranial C6 glioma showing prominent lactate and lipid peak in the tumor (red mark) which is absent in normal brain (blue mark) indicating highly acidic tumor microenvironment. (**E**) *In vivo* drug release of new wafers prepared by changing the polymer composition (W6-W11), tested in C6 glioma model.

As an initial screening, *in vitro* drug release studies were conducted with 20 wt% TMZ loaded nano wafers (W1 to W6) in artificial CSF (Fig. 2A). Wafer with lactic to glycolic acid ratio of 50:50 (W1) showed a burst release of 80% within the initial hours. Increasing the lactic acid content to 75% and 85% reduced the burst release to 54% in W2 and 47% in W3, followed by 100% release in 30 days. This sustained release was further improved by increasing the molecular weight of PLGA to 100–150 kD and PLA content to 5 and 7% in W4 and W5 respectively. However, ~18% burst release was observed within first few hours. At this stage we have introduced 2% PCL (MW 100 kD) with PLGA: 85:15 and PLA (W-6), which showed excellent control on burst release. Considering this, we took W6 for the *in vivo* brain drug release study in rat models.

### *In vivo* drug release from nanofibers in rat models

W6 was implanted by stereotaxic surgery into the cortex region of healthy rat brain and the drug release at different time intervals was examined. Compared to the *in vitro* data, only marginal difference in the drug release pattern was observed in healthy brain. However, when this wafer was implanted in the tumor model, significant burst release (75%) was registered within 24 h (Fig. 2B). Postmortem of the implanted tumor after seven days showed almost complete degradation of the implant in the tumor tissue (Fig. 2C). In sharp contrast, the *in vivo* biocompatibility studies of the same wafer conducted in healthy rat brain showed insignificant degradation even after three months of implantation. To understand this differential effect in tumor versus healthy brain, *in vivo* NMR spectroscopy of both these regions were conducted (Fig. 2D), which showed that the tumor microenvironment was relatively acidic probably due to the elevated levels of lactic acid contributed by anaerobic tumor metabolism. However, *in vitro* drug-release studies under acidic pH was not showing significant burst release or abrupt degradation of fibers, indicating that in addition to acidic pH, numerous tumor enzymes, necrotic fluid, chemokines, cytokines and tumor associated immune cells have contributed to the accelerated degradation of nanofibers. This reiterates that drug-release studies using *in vitro* or even healthy *in vivo* animal models has limited significance, rather more meaningful data using actual brain tumor model is required. Thus, we re-optimized the nanofiber composition for controlled and prolonged release by testing the samples directly in the tumor microenvironment. Accordingly, new wafer compositions were prepared with varied polymer blends (PLA-PLGA-PCL), molecular weights, drug loading ratio (5–30%) and fiber diameter (W6 -W11, Table S1, Supporting Information) and tested were directly in tumor models. We have optimized a library of nanofibers with *in vivo* tumor drug-release properties varying from very fast (2 h) to very slow (60 days) as shown in Fig. 2E. Although better control on sustained release was achieved by optimizing the polymer composition (W11), the release for initial hours was too slow to achieve sufficient drug dosage in the tumor. Exposure of cancer cells to inferior doses of chemotherapeutics may potentiate drug-resistance mechanisms. Ideally, an implant needs to provide sufficient dose of the drug at a constant rate for prolonged periods from weeks to months. However, as shown in Fig. 2E, the wafers W6-W11 were not able to provide such a constant (zero-order) release in the brain tumor.

**Figure 3:**
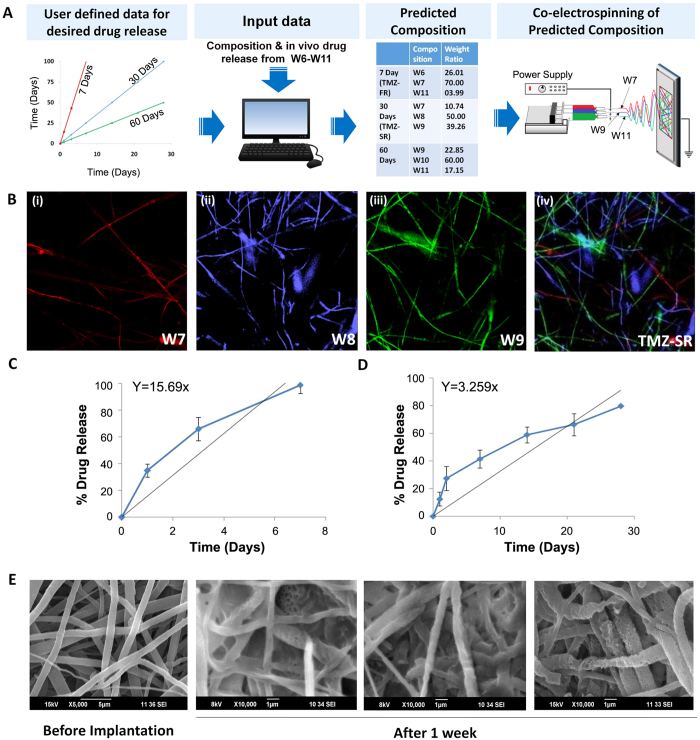
Numerical modelling of nanofiber implants for zero-order release and its preparation using co-electrospinning technique. (**A**) The desired zero-order *in vivo* drug release pattern for 7, 30 and 60 days is represented by three linear curves characterized by their slopes. Actual *in vivo* tumor release data of W6-W11 was fed to numerical algorithm for prediction of fiber composition required for desired release. Numerically predicted nanofiber compositions and their weight fractions for 7 day (TMZ-FR) and 30 day (TMZ-SR) implants were fed to a multi-cartridge electro-spinning station for the co-spinning of predicted polymer-drug compositions. (**B**) Representative confocal images of individual polymer fibers of wafer: (i) W7, (ii) W8 and (iii) W9 and (iv) the final co-electrospun wafer (TMZ-SR) showing combinatorial single implant system formed by individual fibers. (**C**,**D**) *In vivo* drug release pattern of TMZ-FR (7 days) and TMZ-SR (30days) wafers, tested in tumor bearing rat brain. Straight lines indicate predicted release. (**E**) SEM image of TMZ-SR wafer before and one week after the implantation in brain tumor showing differential pattern of degradation in individual nanofibers.

Although, it was tough to achieve zero-order release using bulk eroding polymer compositions, we have optimized a unique method where nanofibers with extremely varied release kinetics (from hours to months) were suitably mixed at appropriate weight fractions and made as a single implant containing separate set of nanofibers to release the drug for a specific period and ‘switch’ the job to next set for another period. This provided an excellent opportunity to tune the drug release over a wide range of periods using these nanofiber libraries. To simplify the practical realization of this concept, we have used a numerical algorithm having conceptual similarities with the ‘color blending technique’ used by the paint manufacturers for creating new colors from a library of primary colors. Here, each polymer nanofiber composition with characteristic *in vivo* drug release pattern in the brain (W6-W11) was pseudo tagged as a specific color and the program was asked to predict a new combination of these colors that may be mixed to obtain a new color (desired drug release pattern). i.e., a new wafer with desired release kinetics was defined by the slope of a zero-order graph for a specific period (e.g. 7, 30 or 60 days) as shown in Fig. 3A(i). Thus, the program predicted nanofiber compositions and weight fractions that are required to be spun together to create a new wafer capable of providing desired release profile. This data was fed into a co-electrospinning unit (Fig. 3A(ii)) loaded with multiple cartridges filled with various polymer-drug blends (W6-W11) to make the wafer having desired drug release profile. The co-electrospinning technique gives excellent opportunity to spin multiple polymer-drug compositions together into a single target and thus creating versatile nanofiber implants with scalability and reproducibility.

For the detailed *in vivo* evaluation and validation of the concept, we have selected two representative implants: TMZ-FR (20 wt% TMZ loaded wafer designed for 7 day release) and TMZ-SR (20% TMZ loaded wafer designed for one month release). In order to differentiate these multiple fibers in the same implant, we labeled them with fluorescent dyes and representative confocal microscopic images (Fig. 3B(i–iv)) show the distribution and morphology of three different nanofibers (W7, W8, W9) that were co-spun to form TMZ-SR. These implants were tested *in vivo* and Fig. 3C and D shows the *in vivo* drug release patterns in orthotopic rat glioma models. Interestingly, these wafers showed sustained and prolonged release for 7 days (TMZ-FR) and 30 days (TMZ-SR) as predicted by the numerical algorithm. TMZ-FR showed ~100% release over 7 days while TMZ-SR registered 41% release by one week, followed by 59% and 80% release in second and fourth weeks respectively, rendering an overall release for 30 days. The slope of the curves showed close correlation with the predicted data suggesting that the *in vivo* release pattern was the culmination of release from individual fibers that were designated for specific time points. Figure 3E shows the SEM images of TMZ-SR wafer after one week of implantation indicating morphological changes, wherein certain set of fibers showed signs of early degradation while others remained largely intact. This suggests that different fibers were degraded at various rates, which correlated with the predicted and observed release patterns. Recently, Yu *et al*., has reported an interesting tri-layered, electrospun polymeric nanofiber system for attaining zero-order release kinetics *in vitro*. Three different layers of polymer-drug composition is formed by triaxial electrospinning of ethyl cellulose where drug loading at different layers were manipulated to obtain zero-order release for 20 h under *in vitro* condition^53^. Compared to this approach, here we show prolonged (30 days) zero-order release under *in vivo* conditions by spinning three different fibers onto a single target, which is easier for independent control during bulk-scale preparation. Effectively, we have shown that by rational selection of combinatorial nanofiber libraries, it is possible to achieve controlled drug release in brain-tumor even with bulk-eroding polyester nanofibers. Although we demonstrated results for TMZ, this method can be used as a platform technology for other chemotherapeutic agents such as BCNU, paclitaxel, etc. The composition of the polymer blends needs to be re-optimized for desired release profile based on the chemical structure and hydrophobic/hydrophilic characteristics of the candidate drugs.

### *In vivo* brain biodistribution versus systemic leakage

**Figure 4:**
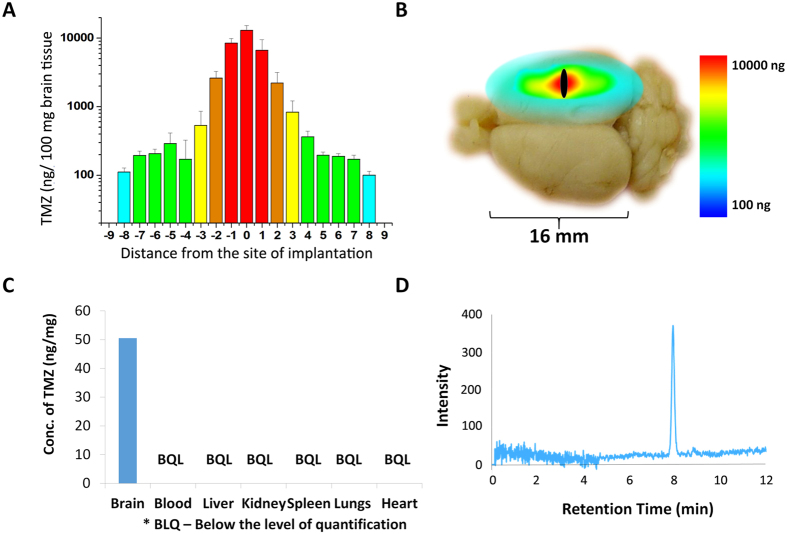
Distribution of TMZ in rat brain vs. other organs and systemic circulation. (**A**) Brain drug distribution (penetration) of TMZ released from TMZ-FR wafer at 48 h and (**B**) the colour coded brain map representing TMZ penetration (8 mm) throughout the wafer implanted cerebral hemisphere. (**C**) Graph showing amount of TMZ present in brain versus plasma and other vital organs, 48 h after implantation of TMZ-FR wafer showing no detectable TMZ either in circulation or organs. (**D**) HPLC chromatogram showing detection of 100 ng/ml TMZ, when spiked in serum.

In addition to the controlled release, it was critical to achieve localized drug distribution in the brain without systemic leakage and toxicity. Brain-distribution of TMZ released from TMZ-FR (size: 5 × 1 mm, weight: 18 mg, TMZ dose: 3.6 mg) by HPLC shows (Fig. 4A) drug-diffusion up to 8 mm from the site of implantation. Corresponding color coded brain map is shown in the Fig. 4B. One of the main limitations of the clinically used Gliadel wafer was tumor recurrence, which was almost inevitable in all the patients. Studies have reported that in 90–95% of cases, recurrence happens within 2 cm margin of the tumor resected cavity. Hence, effective prevention of recurrence needs 2–3 cm drug diffusion from the implanted site. In the present case, TMZ diffusion from the nanowafer could be detected up to 8 mm by 48 h towards each side of the cerebral hemisphere. This resulted in drug diffusion covering the entire hemisphere of cerebrum where wafer was implanted, depicted in Fig. 4B. Diffusion beyond 8 mm could not be assessed due to the limited size of rat brain. A higher animal model (pig) needs to be tested for measuring higher diffusion. However, systemic leakage, tested by HPLC for 0–30 days, showed no detectable drug dose either in serum or other vital organs such as liver, kidney, spleen, heart and lungs (Fig. 4C). The detection limit of HPLC for TMZ was 100 ng, estimated by spiking the serum *ex vivo* is shown in Fig. 4D. This means that, the released drug was confined well within the 8 mm brain tumor area around the implanted site with little systemic leakage. This is a significant data indicating that the nanofiber implants can avoid systemic toxicity faced by current oral dosage of TMZ (150–200 mg/m^2^/day).

### Engineering the theranostic property in nanofiber implant for MRI guided implantation and monitoring

**Figure 5:**
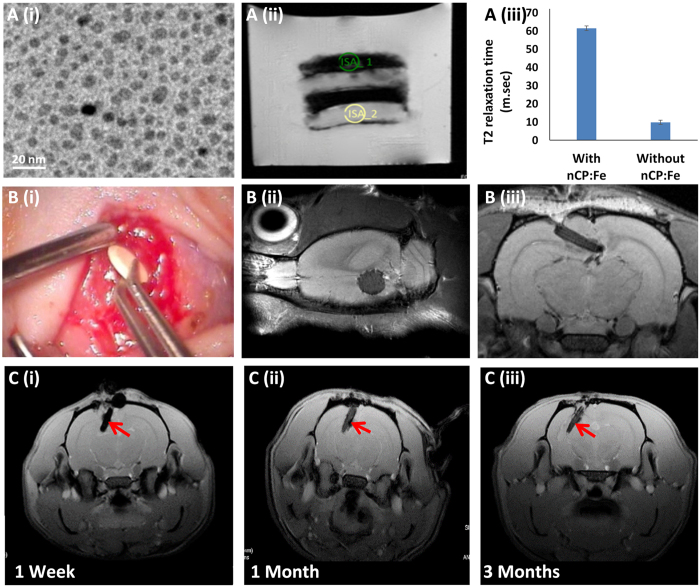
Engineering the theranostic property in nanofiber implant for MRI guided implantation and monitoring. (**A**(i)) TEM image of ~10–15 nm size nCP:Fe nano-contrast agent used for the preparation of theranostic nanofiber implant (**A**(ii)) MR image showing enhanced T2 contrast (dark) in nCP loaded wafer compared to the control wafer (light). (**A**(iii)) Graph showing change in T2 relaxation time between the wafers loaded with and without the contrast agent, nCP:Fe. (**B**(i)) Photograph and (**B**(ii,iii)) T2 weighted MR images of TMZ-SR wafer implanted in the rat brain cortex. (**C**) Shows T2 weighted MR images of wafer implanted rat brain, imaged up to 3 months.

One of the major limitations of currently used polymeric biomedical implants is their inability to provide sufficient contrast (magnetism or x-ray absorption) for medical imaging methods such as MRI or CT. Once implanted in the brain, it is desirable to have non-invasive imaging of the implant to estimate acute toxicity, edema, inflammation, immune reactions, implant degradation and brain clearance. To address this, we have conferred MR contrast to our nanofiber implant by co-loading a novel MR contrast agent, Fe^2+^ doped calcium phosphate (nCP) nanoparticles, which are being developed in our lab for MR/CT guided nano-theranostics^54^,^55^. Being an endogenous bio-mineral content of our body, calcium phosphate is biocompatible than any other engineered nanoparticle contrast agents. Iron being an important elemental component in the serum, it is safe to use as a dopant ion in nanoparticles^56^. We used ~50 μg/kg body weight of Fe^2+^ in the total implant which is well within the tolerable limit. Fig. 5A(i)) shows the TEM image of ~10–15 nm size nCP:Fe nano-contrast agent used for the preparation of theranostic nanofiber implant. SEM analysis revealed non-beaded micro-morphology of the theranostic wafer made of smooth nanofibers ((Figure S3, Supporting Information). The MR images of wafers (Fig. 5A(ii))) with nCP:Fe loading(ISA 1) showed enhanced *T2* contrast (dark) compared to the control wafer without nCP:Fe ISA 2) making it clearly visible in the MRI. T2 map (Fig. 5A(iii)) also shows significant reduction of transverse magnetic relaxivity from 61.56 ± 1.3 to 9.95 ± 1.18 m. sec in nCP:Fe loaded wafer, indicating its suitability for *in vivo* MR imaging. Thus, we achieved a theranostic nanofiber implant for image-guided implantation and non-invasive monitoring.

### *In vivo* brain compatibility and systemic toxicity effects of nanofiber implant in rat

Next, we have studied maximum tolerated dose (MTD) for TMZ loaded wafers in rat brain. Maximum two wafers, each weighing 17.5 mg, could be implanted due to the limited brain volume in the rat. The MTD was not reached up to maximum tested dose of 7 mg TMZ. In addition, we tested systemic toxicity by monitoring different haematological parameters in TMZ-SR wafer implanted animals compared to untreated control at different time intervals up to 3 months. The data (Table S2, Supporting Information) showed no sign of haematological toxicities including leukopenia or thrombocytopenia in TMZ-SR implanted group compared to untreated or bare wafer. Further, histopathological examination of liver, kidney, lungs, spleen and heart tissues showed no pathological changes in any of the study groups indicating absence of any organ toxicity by wafer implantation (Figure S4, Supporting Information). This observation correlated with the fact that the systemic leakage of drug from the brain implanted wafer was insignificant to cause any systemic damage. In effect, it shows that even after one month of continuous drug release in brain, no serious systemic leakage or toxicity was observed in the animal.

Next, the biocompatibility assessment of bare and drug loaded nanofiber implants were studied in healthy rat brain for up to three months. Samples were implanted in the cortical region of brain as shown in Fig. 5B(i). T2 weighted MR images show the circular implant in the brain (Fig. 5B(ii,iii)). Body weight changes registered a dip in the initial days after surgery in all animals including sham control, nonetheless, they regained weight and normalized subsequently (Figure S5, Supporting Information). Brain edema is a frequent adverse event in the clinics after implantation of drug-loaded wafers in glioma patients^57^. Fluid accumulation due to edema will normally give bright *T1* contrast at the implanted site, however, we have not observed such event in the present case as seen in the MR images recorded at different time points, up to three month (Fig. 5C(i–iii)). The implant was found compatible with the adjacent brain tissue with no sign of inflammation at the implant-tissue interface up to the study duration of three months. The animal recovered usual aesthetics and maintained normal behavioral pattern for the entire study period as shown in video footage (Videos S1 and S2, Supporting Information).

**Figure 6:**
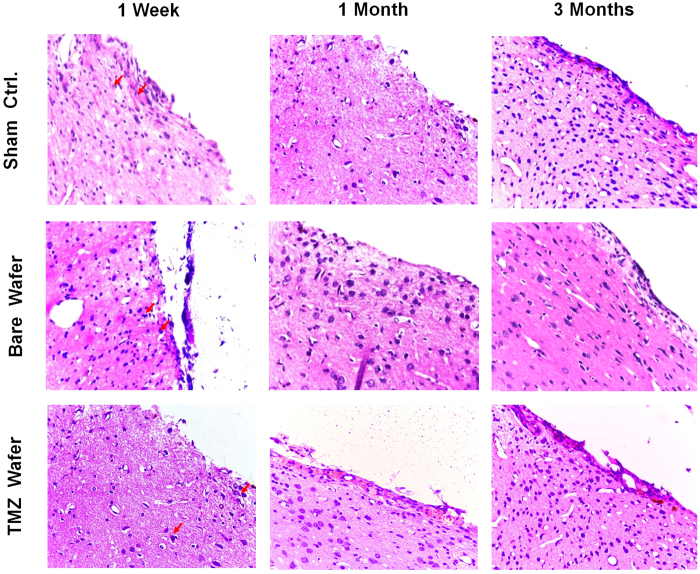
Brain biocompatibility of nanofiber wafers. Histopathology sections of rat brain-wafer interface in sham control, bare wafer and TMZ-SR wafer implanted animals: Minor inflammation by way of marginal elevation in the leukocyte count (red arrow heads) was seen after one week; however, the same was reduced by 1–3 month.

Histology of the coronal sections of implant–brain interface taken at various time points are shown in Fig. 6. Both bare and TMZ loaded implants showed minor inflammation, indicated by the slight elevation of leukocyte count, immune cell infiltration and thickening of the tissue at the implant-tissue interface. This may be due to the compression of brain tissue caused during the implantation of wafer in intact brain. In clinical scenario, this situation may be different as the wafers will be implanted in a cavity formed by tumor debulking. Further, an array of both pro-inflammatory and anti-inflammatory cytokines (IL-1α, IL-6, IL-4, IL-10, IL-2 and IFN-γ) at different time intervals after implantation (72 h to 3 months) were studied to elucidate the immunological reaction to the implant (Figure S6, Supporting Information). Although a slight increase in the pro-inflammatory cytokines was observed by 72 h post implantation, overall levels remained within the normal limits. Effectively, this three month study indicated that the wafer caused no serious pathological changes or adverse immunological response in the rat brain.

### Anti-tumor effects of TMZ wafers in glioma model: *in vitro* and *in vivo* studies

Initial testing of anti-cancer efficacy of TMZ released from the nanofiber implant was tested against glioma cells, *in vitro.* At equivalent concentration, TMZ loaded wafers showed enhanced cytotoxicity (81.5%) compared to free-TMZ (50.5%) or bare wafer (4.6%) towards glioma cells, as estimated by MTT, apoptosis, live-dead assays and electron microscopy (Figure S7, Supporting Information). The enhanced cytotoxicity effect may be attributed by the sustained release of fresh TMZ from the nanofibers, which was not in the case of free-drug treated cells, where the whole drug was exposed to the culture medium from time-zero. We found that almost 100% of free TMZ was degraded by 9 h in culture medium at 37 °C whereas ~80% of the polymer fiber encapsulated TMZ remained intact in the implant as shown in the HPLC data (Figure S8, Supporting Information).

**Figure 7:**
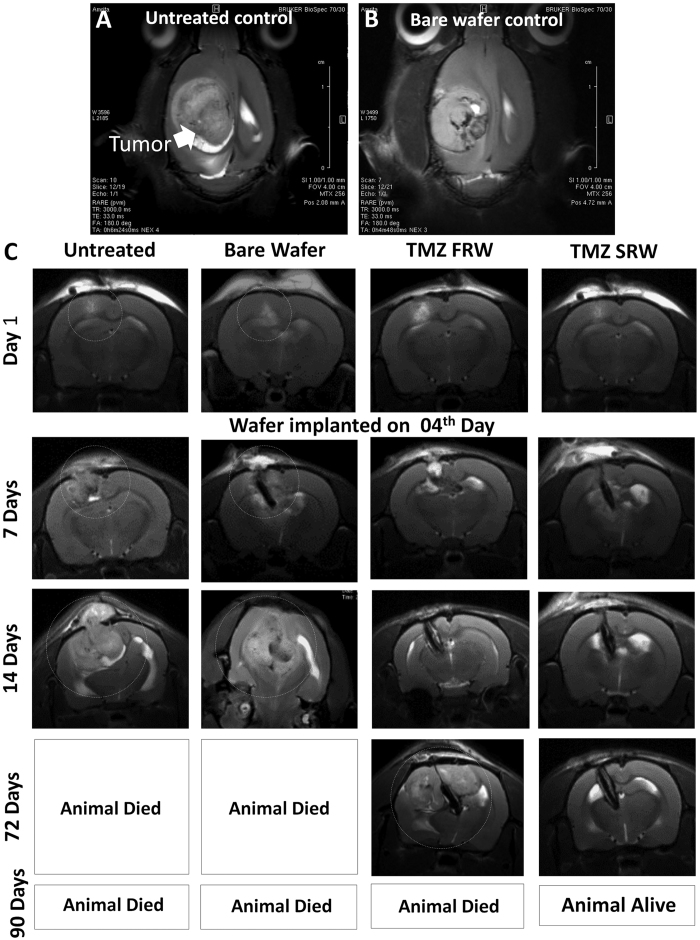
MRI based assessment of temozolomide loaded nanofiber implant therapy in orthotopic C6 glioma model. (**A**) T2 weighted MRI images of full grown C6 glioma in untreated and placebo control group, showing the aggressive nature of tumor occupying a major portion of brain hemisphere. (**B**) MRI image of tumor implanted with bare wafer (no drug). (**C**) Representative MR images of day 1–90 for the untreated, bare-wafer, TMZ-FR and TMZ-SR wafer implanted animals. Significant tumor growth can be seen in untreated and bare wafer groups by day-14. TMZ-FR showed initial reduction in tumor growth, but recurrence happened in 54% animals leading to tumor re-growth by day-72. TMZ–SR showed prolonged control on tumor growth for the entire study period.

Next, the anti-tumor efficacy of both fast releasing (TMZ-FR) and slow-releasing (TMZ-SR) implants were studied in orthotopic C6 rat glioma model. The first-line treatment of malignant gliomas include maximal surgical resection of the tumor. The local drug delivery wafers (Gliadel) are placed into the resected tumor cavity to manage the left-out tumor cells, which are inaccessible or non-resectable. To mimic this scenario of residual tumor cells, first we injected 5 × 10^5^ glioma cells in to the rat brain cortex on day 0. After 3 days, with the help of MRI guidance, a surgical cut was made in the exact location of tumor cell injection and wafers with nearly 3.5 mg of TMZ (equivalent to current oral dose) were implanted. Thus the wafer was implanted neither in a full grown tumor nor the healthy brain. Animal behaviors were closely monitored and tumor volume changes were tracked using MRI up to 90 days. Figure 7A and B shows the representative MR images (sagittal sections) of C6 glioma in untreated and bare-wafer controls after 14 days, indicating aggressive tumor growth with no placebo-effect. Figure 7C shows aggressive tumor growth by day-14 in untreated control animals. The tumor was growing even towards the exterior region of the brain through the skull opening that was created for cell injection. The median survival for this control group was 24 days. Whereas, in case of TMZ-FR and TMZ-SR, significant control on the tumor growth was observed up to two months. However, most surprisingly, after 60–72 days ~57.14% of animals treated with TMZ-FR showed tumor recurrence from the region adjacent to the primary tumor or the site of implantation (Fig. 7C, 72 day data). In sharp contrast, 85.7% animals in TMZ-SR group remained tumor free for the entire study period of 90 days with no sign of tumor recurrence (Fig. 7C, TMZ-SRW).

**Figure 8:**
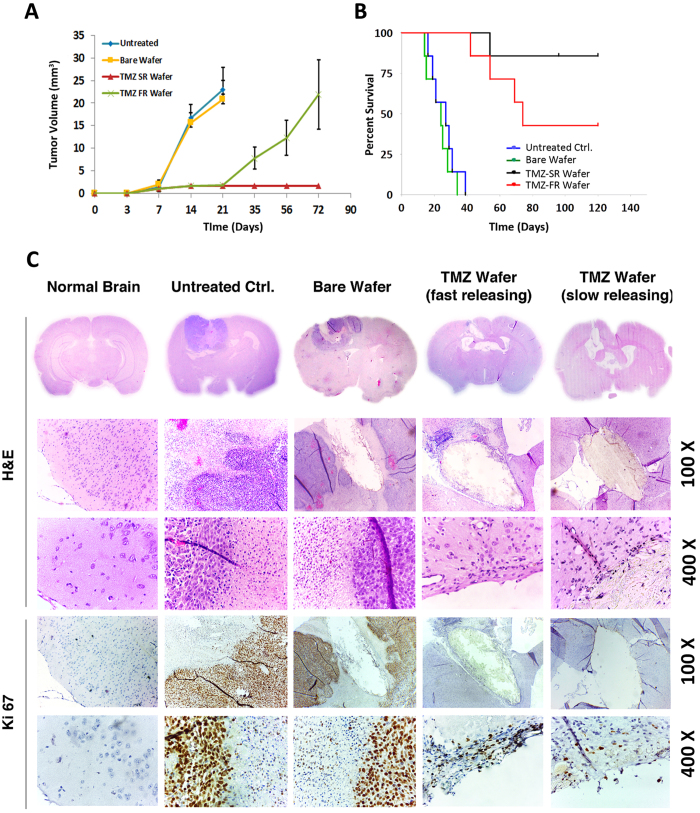
*In vivo* tumor reduction ability of TMZ wafers in orthotopic C6 glioma model. (**A**) Graph showing quantitative tumor volume reduction in orthotopic glioma in different treatment groups, calculated from MRI. (**B**) Kaplan-Meier survival curve showing survival of animals in different treatment groups. ***P < 0.0001. (**C**) H&E (upper three panels) and Ki67 staining (lower two panels) of brain sections of different treatment groups at day 14.

Quantitative estimate of tumor volume changes determined using MRI over 72 days (Fig. 8A) clearly reflects the tumor recurrence in TMZ-FR treated animals versus TMZ-SR group. Survival advantage, studied using Kaplan Meier method (Fig. 8B) showed significant improvement in TMZ-SR group compared to TMZ-FR and control groups. While the untreated and placebo controls registered median survival of 24 and 27 days (P value = 0.4156) respectively, TMZ-FR showed 74 days (P value = 0.0001) and median survival was not reached in the case of TMZ-SR implanted animals (P value = 0.0001) as 85.7% remained long term (>90 days) survivors. Evidently, one month sustained release of drug from TMZ-SR showed significant advantage over 7-day releasing wafer TMZ-FR in inhibiting the tumor recurrence and improving survival. This result is significant compared to the latest report on TMZ loaded Gliadel-type wafers (5 mg TMZ, one week release in 9L gliosarcoma) showing median survival advantage of 28 days with 25% long term survivors^35^. By doubling the dose to 10 mg, the same wafer showed 37.5% long-term survivors. In comparison, the present data suggests that by increasing the sustained release to 30 days using rationally selected combination of bulk-eroding polyester nanofibers, the long-term survival could be increased to 85.7% even with low-dose (3.5 mg) TMZ. The result may be further improved with increase in drug dosage to 7 or 10 mg. Histopathology studies on brain samples collected from animals sacrificed on 14^th^ day following tumor inoculation is shown in Fig. 8C. Coronal sections of rat brain from different treatment groups were compared after staining with H&E and Ki67. In both untreated and placebo controls, large tumor was seen occupying major portion of the brain cortex, and characterized by very closely packed small C6 glioma cells. Ki67 staining confirms aggressively growing tumor regions having sharp interface with healthy tissue. On the other hand, no tumor growth was seen in both the TMZ-FR or SR wafer implanted animals, showing effective anti-tumor activity of the wafers. Nevertheless, few Ki67 positive proliferating cells were seen in the margins of TMZ-FR implanted site (image with 400X magnification), and this could be correlated with the incidence of tumor recurrence. In contrast, only very few Ki67 positive cells were seen on TMZ-SR treated animals and that could be eliminated by further sustained release of TMZ from the wafer, preventing the recurrence. This result suggests that in addition to localized drug delivery of chemotherapeutics within brain, prolonged release for more than a week is critical for the effective inhibition of aggressive tumor growth and prevention of recurrence.

## Conclusions

We have demonstrated a unique method to create flexible, polymeric nanofiber implant for prolonged and sustained release of anti-glioma drug, Temozolamide, in rat glioma model. We used three well known bulk-eroding polymers, PLGA, PCL and PLA and created a library of nanofiber blends with *in vivo* drug release kinetics in brain varied from too fast (hours) to too slow (months). Using a conventional color blending algorithm, suitable nanofiber combination with optimum weight fractions required for zero-order drug release for specific periods were predicted and prepared by co-electrospinning technique, and tested *in vivo* brain tumor models. Effectively, by combining different fibers into single implant, we achieved fiber-by-fiber controlled drug release for extended duration up to 30 days in a challenging tissue micro-environment like brain tumor. The importance of prolonged drug release in controlling tumor growth and prohibiting tumor recurrence has been demonstrated in orthotopic brain tumor models, wherein >85% of wafer implanted animals showed prolonged survival of more than three months. The materials and methods used for the preparation, prediction, and optimization are simple, scalable and clinically translational. This method could open–up new opportunities to achieve sustained and prolonged release of many other anti-glioma drugs using implantable nano-systems made from combinatorial library of bulk eroding polymers having known *in vivo* pharmacokinetics.

## Methods

### Materials

The polymers used for the electrospinning, PLGA 50:50 (45 kD), 75:25 (75 kD), 85:15 (75 kD), PLA (150 kD) and PCL (100 kD) were purchased from Sigma Aldrich, Goodfellow Cambridge Ltd., England and Polysciences, USA. Temozolomide was procured from A K Scientific, USA. Methanol, Glacial acetic acid and Chloroform, purchased from Merck were of HPLC grade. All other materials and reagents used were of analytical grade.

### Fabrication of TMZ loaded PLA-PLGA-PCL electrospun wafers

A library of 20 wt% loaded TMZ wafers (W1 to W11) was made by electrospinning technique using eleven different polymer blends. The polymer blends were made by varying PLGA:PLA:PCL ratios, in which PLGA was varied form 50–100%, PLA from 5–50% and PCL from 1–10% (*wt/wt*). The polymer blend solutions were first made by dissolving individual polymers in acetone (PLGA) or chloroform (PLA and PCL), mixed and blended further for 4 h. TMZ was then added to the polymer blends and allowed to mix for 4 h with intermittent sonication. Each TMZ–polymer solution was taken in a 10 ml syringe fitted with a 21 gauge blunt end needle and pumped at a rate of 0.25–3 ml/h using a syringe pump (KD Scientific, USA). Once a high voltage difference of 12–18 kV was applied between the nozzle and the grounded collection target, the polymer jet breaks up into fibers from the Taylor Cone and the solvents were evaporated, leading to the formation of relatively dry fibers, subsequently collected in the aluminium foil to obtain multi-layered fiber mats. In order to vary the nanofiber diameter in different wafers, the electrospinning parameters including needle diameter, flow-rate, tip-target distance, voltage were varied. For preparing nanofibers low weight percentage (wt%) polymer solutions were used (less viscosity) along with higher gauge needle and more tip-target distance. Micro fibers were made by using high weight% polymer solutions, smaller gauge needle and lesser tip-target distance. The final theranostic TMZ wafer for 7 day (TMZ-FR) and 30 day (TMZ-SR) drug release were made by co-electrospinning, each using three different polymer blends loaded with TMZ and 0.5 w% nCP:Fe and chosen by numerical algorithm from the library (W6–11). The polymer blend ratios for final wafers were W6:W7:W11 at 26:70:4 for TMZ-FR and W7:W8:W9 at 11:50:39 for TMZ-SR.

### Characterization of co-electrospun PLA-PLGA wafers loaded with TMZ

The wafers were characterized for their size, surface morphology and microfiber distribution using scanning electron microscope (SEM; JSM-6490 LA, Japan). TMZ distribution pattern in the nano-fibers in term of elemental distribution of Nitrogen were determined by Energy-Dispersive spectroscopy (EDS). X-ray diffraction pattern of 20 wt% TMZ loaded wafer, pure TMZ, bare wafers and were analyzed by X’Pert PRO (PANalytical B. V., The Netherlands). NMR spectra of TMZ in free form and in wafer loaded state was taken after dissolving sample in DMSO, using 9.4 Tesla Bruker Avance III, 400 MHz FT NMR Spectrometer.

### Determination of encapsulation efficiency and drug loading

Known quantity of TMZ- wafers was taken, dissolved in chloroform and the drug was separated using methanol. The TMZ content was analyzed using Shimadzu HPLC system (CBM-20 A) equipped with Qualisil gold C-18 column (4.6 × 250 mm, 5 μ) and a PDA detector (SPD-M20A). A mobile phase containing 0.5% glacial acetic acid and methanol (1:1 v/v) was used at a flow rate of 1.0 ml/min. Drug concentration was estimated using UV absorption at 330 nm (retention time 7.8 min).


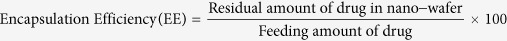


### *In vitro* drug release kinetics

5 mg of TMZ-loaded wafer was immersed in releasing media [artificial CSF (119 mM NaCl, 26.2 mM NaHCO_3_, 2.5 mM KCl, 1 mM NaH_2_PO_4_, 1.3 mM MgCl_2,_ 10 mM glucose, pH 7.4] in triplicates inside a shaking incubator set at 60 rpm and 37 °C. The releasing media were replaced with fresh media at definite time intervals and analyzed for TMZ using HPLC as described above.

### Cell culture

U87MG and C6 glioma cell lines were procured from National Centre for Cell Sciences, Pune, India and cultured in Minimum Essential Medium (MEM) and Dulbecco’s Modified Eagle Medium (DMEM) respectively, supplemented with 10% fetal bovine serum; 100 IU/ml penicillin and 100 μg/ml streptomycin (all reagents from Gibco, Invitrogen), in a humidified atmosphere of 5% CO_2_ at 37 °C.

### Determination of *in vivo* drug release and drug distribution

*In vivo* drug release kinetics and brain drug distribution of TMZ loaded wafers were studied in healthy and tumor induced Wistar rats. Animals were implanted with TMZ wafers in the brain using aseptic surgery. Rats were anesthetized by i.p injection of ketamine and xylazine and prepared for aseptic surgery by shaving the head followed by sterilization using ethanol and povidine-iodine solution. 1.5 cm long skin incision was made lateral to midline, periosteum was carefully elevated and a small craniotomy of 2 × 8 mm was made. A small incision was made in the dura and brain using a micro-scissors and wafer disc of 5 mm dia. was carefully implanted into the brain. Hemostasis was achieved using Surgicel^®^. The skin incision was sutured using 4-0 Vicryl (Johnson&Johnson) and animals were critically monitored until recovery. All animal procedures were approved by the institutional animal ethics committee, Amrita Institute of Medical Sciences & Research Centre and was done according to CPCSEA (Committee for the Purpose of Control and Supervision of Experiments on Animals, Govt. of India) guidelines. The animals were sacrificed at predetermined time intervals (1, 3, 7, 14, 21 and 28 days; n = 3) and the wafers were retrieved to determine the remaining TMZ content using HPLC. Tissue and blood samples were also collected and analyzed for the presence of TMZ. In order to determine the brain drug diffusion from the implanted wafer, the rat brain was collected after 48 h of implantation and sliced into 1 mm thick sections using rat brain matrix (coronal, ASI instruments, USA). Each slices were thoroughly homogenized using Miltenyi gentlemacs dissociator (Miltenyi Biotech, Singapore) and protein precipitation was carried out using acetone. The protein separation was carried out overnight incubation followed by high speed centrifugation. The drug containing supernatant was concentrated using Centrivap vacuum concentrator (Labconco, USA) and TMZ quantification was done using HPLC.

### *In vivo* biocompatibility of nano-polymeric wafers

54 healthy adult Wistar rats were subdivided into nine groups (1 week, 1 month, and three months for sham control, placebo wafers and TMZ loaded wafers) with 6 rats in each group. Animals were implanted with wafers using aseptic surgery as described above. Animals were closely monitored for behavioral changes, abrupt neurological deficits and weight loss associated with toxicity. *T1* weighted and *T2* weighted MRI images of the brain were taken at different time intervals to find out any incidence of edema around the implanted area. Peripheral blood was collected to check serum cytokine levels using (BD Cytometric Bead Array (CBA), BD Biosciences, CA, USA). Brain and other important organs were collected from each animal after a desired period of time to study the biocompatibility and toxicity of nanofiber wafers. The tissues were fixed using formalin, embedded using wax, sectioned and detailed histopathology analysis (H&E staining) was carried out to study the wafer biocompatibility.

### Magnetic Resonance Imaging of animals

The MR images of brain were taken using 7 T animal MRI station (70/30 USR Bruker Biospec, USA). The T1weighted images of rat brain were acquired by using FLASH T1 echo sequence and T2weighted images were acquired by using RARE T2 echo sequence. The mean signal intensity values were calculated in the region of interest.

### *In vivo* antitumor activity assessment in orthotopic C6 glioma models

The antitumor activity assessments of TMZ-FR and TMZ-SR wafers were carried out in intracranial C6 glioma model. 5 × 10^5^ cells C6 glioma cells were implanted in the brains of female Wistar rats (8–12 week old), 0.5 mm anterior and 3 mm lateral to the bregma using steriotaxic equipment (ASI Instruments, USA). These animals were randomly divided into four groups (7 animals per group): (i) untreated control, (ii) bare wafer, (iii) TMZ-FR and (iv) TMZ-SR. The wafer implantation was carried out on the fourth day from tumor inoculation. The wafers were carefully implanted into the tumor bed after MRI images assisted tumor localization. Animal behaviors were closely monitored each day and the tumor growth was assessed using MRI. Separate set animals were maintained for the histopathology analysis. The brain samples collected on the 21^st^ day, fixed using formalin, embedded using wax, sectioned and standard histopathology analysis (H&E staining) and Ki67 staining were carried out to study the anti-tumor activity.

### Statistical Analysis

All results were represented as mean or mean ± standard deviation. Student’s t-test and ANOVA were used respectively to evaluate the significance in two or multiple groups. Significance between groups in the Kaplan-Meier survival analysis was determined by Chi-square test.

## Additional Information

**How to cite this article:** Ramachandran, R. *et al*. Theranostic 3-Dimensional nano brain-implant for prolonged and localized treatment of recurrent glioma. *Sci. Rep.* **7**, 43271; doi: 10.1038/srep43271 (2017).

**Publisher's note:** Springer Nature remains neutral with regard to jurisdictional claims in published maps and institutional affiliations.

## Supplementary Material

Supporting Information

Supplementary Video S1

Supplementary Video S2
