# Supplementary material for: Theranostic 3-Dimensional nano brain-implant for prolonged and localized treatment of recurrent glioma
Source: Sci Rep. 2017 Mar 6;7:43271. doi: 10.1038/srep43271 (PMC5338016; doi:10.1038/srep43271)
Supplement: Supporting Information [file srep43271-s1.pdf]

Supporting Information

Theragnostic 3-Dimensional nano brain-implant for prolonged and localized treatment of recurrent glioma

Ranjith Ramachandran, [1]Vijayabhaskar Reddy Junnuthula,[1] G Siddaramana Gowd,[1] Anusha Ashokan,[1] John Thomas[1], Reshmi P,[2] Anoop Thomas,[3]Ayalur Kodakara Kochugovindan Unni[2] Dilip Panikar[3#], Shantikumar V Nair[1] and ManzoorKoyakutty[1]**

[1]Amrita Centre for Nanosciences and Molecular Medicine
[2] Central Lab Animal Facility, Amrita Institute of Medical Sciences& Research Centre
[3] Department of Neurosurgery, Amrita Institute of Medical Sciences& Research Centre
Amrita Vishwa Vidyapeetham, Amrita University, Kochi, 682041, Kerala, India,

Methods

In vitro cytotoxicity assays

Cytotoxic potentials of TMZ loaded electrospun wafers were studied using MTT assay. Briefly, 1x10^5 U87MG cells were seeded in T75 flasks and incubated for 24 h. After incubation the media were replaced and added with 5 mg of TMZ-FR wafers. Bare wafer and pure drug treated control groups were also maintained. Cell viability after 96 h was determined by MTT assay (Sigma, St. Louis, USA). For determining cell growth response, bare and TMZ loaded wafers were seeded with 2 x 10^4 cells and allowed to attach and grow for 96 h. SEM imaging of the scaffolds were taken after gluteradehyde fixation (2%). The functional validation of the same was done using live dead assay. The cells after treatment with different wafers were allowed to incubate with calcein-AM and ethidium homodimer-1 mix as per manufacturer's instructions (Life technologies ™, Invitrogen). The wafers were then imaged for the green fluorescence of calcein-AM (indicating live cells) and red fluorescence of ethidium homodimer-1 (indicating dead cells) using a confocal microscope (TCS SP5, Leica Germany). For determining the mechanism of cytotoxicity, 2.5 x10^5 cells were treated with TMZ wafer, bare wafer, camptothecin (positive control) and media (negative control). The cells were harvested after 96 h, washed and treated with Annexin V FITC and PI cocktail as per manufacturer's instructions (Invitrogen, Cat. No. V13245) and analyzed using flowcytometer (FACS Aria II, BD USA).

Systemic side-effect assessment of TMZ loaded PLA-PLGA wafers

Blood samples were collected at 1 and 3 months after wafer implantation (bare or TMZ-wafer). The hematological parameters (Total counts, Differential Counts and Platelets) were analyzed to determine the systemic toxicities.

Results

Table S1. Different polymer blends used for making nano-fiber library.

Sample ID	Composition	Glycolide: Lactide: Caprolactone ratio	Drug Loading (wt%)	Voltage (kV)	Flow rate (ml/h)	Humidity (%)	Average Fibre Dia.(nm)	Encapsulation Efficiency (%)	Effective Drug loading (wt %)
W1	PLGA 50:50	1:1:0	20	18	0.75-1	55-60	400-600	96.6	19.3
W2	PLGA 75:25	3:1:0	20	18	0.75-1.25	55-60	400-600	92.7	18.5
W3	PLGA 85:15	5.6:1	20	15	1.5-2	55-60	400-600	96.5	19.3
W4	PLGA :PLA	6.1:1:0	20	15	1.5-2	55-60	800-1000	94.2	18.8
W5	PLGA:PLA	8.1:1:0	20	18	1-1.5	55-60	800-1000	95.9	19.2
W6	PLGA:PLA:PCL	6.7:1:0.5	20	15-18	1-2	55-60	800-1000	94.4	18.9
W7	PLGA:PLA:PCL	9:1:1	20	15-18	1-2	55-60	800-1000	95.7	19.1
W8	PLGA:PLA:PCL	10.1:1:1	20	15-18	1-2	55-60	800-1000	94.1	18.8
W9	PLGA:PLA:PCL	21.8:2.2:1	20	15-18	2	55-60	800-1000	92.9	18.6
W10	PLGA:PLA:PCL	30.7:1:1.7	20	15-18	2	55-60	800-1000	92.7	18.5
W11	PLGA:PLA:PCL	10.2:1:1.2	20	15-18	1.75-2.5	55-60	800-1000	94.5	18.9

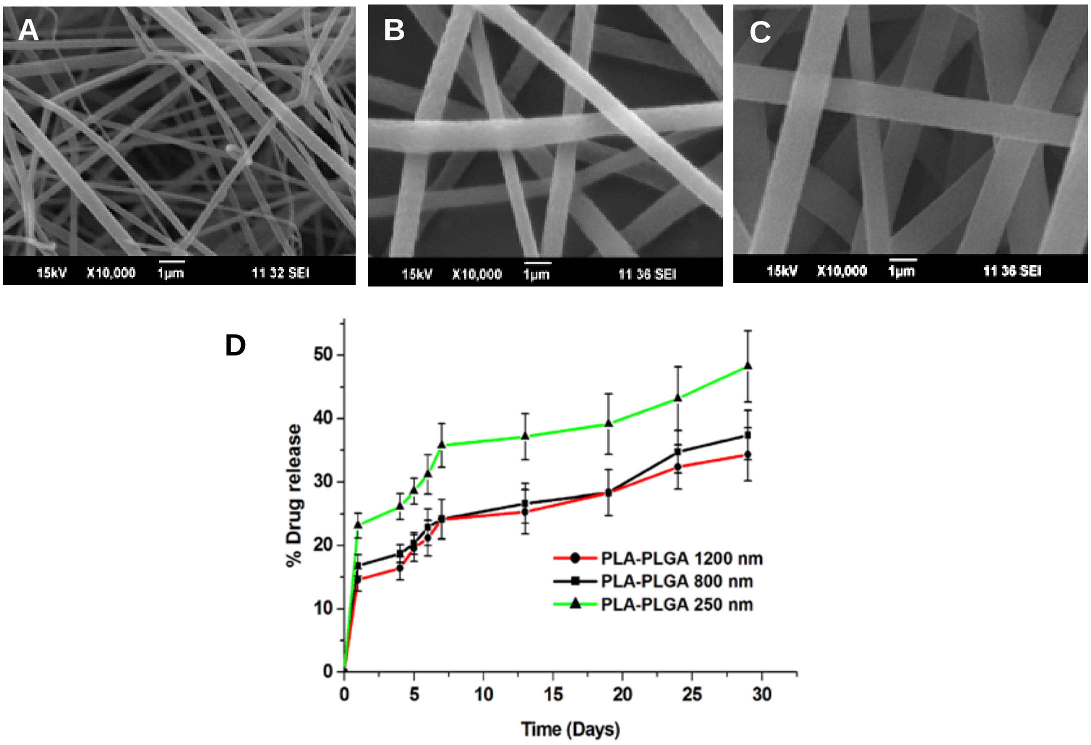

Figure S1 Effect of fiber diameter on TMZ release. SEM images of 20 wt% TMZ loaded wafers having average fiber diameter of (A) 250 nm, (B) 800 nm and (C) 1200 nm. (D) Graph showing the *in vitro* release profile of 20 wt% TMZ loaded wafers. The wafers made of 250nm nano-fibers released TMZ faster compared to 800 and 1200 nm fibers.

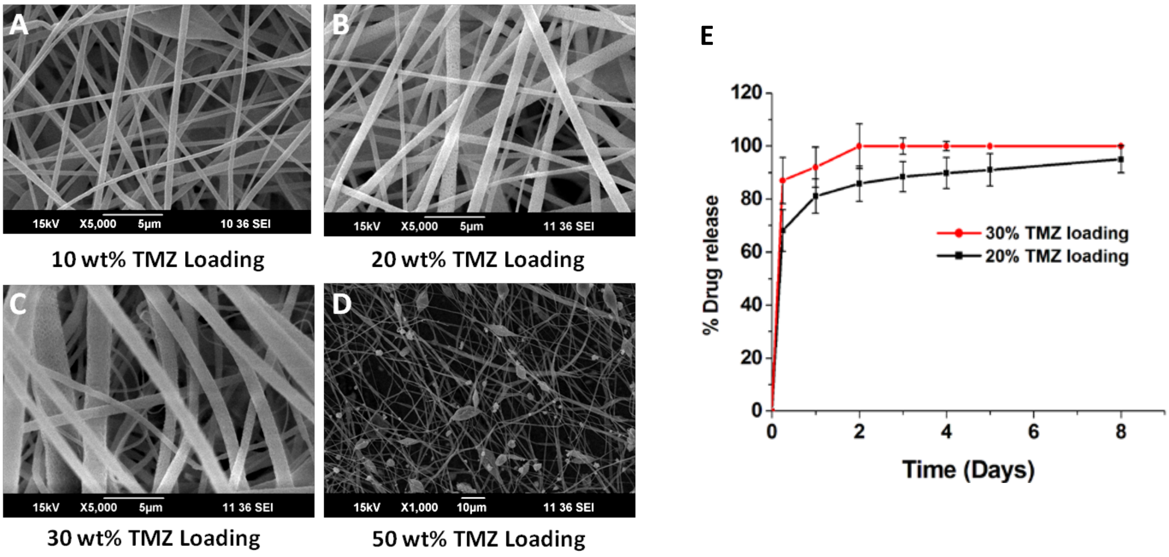

Figure S2 A-D) SEM images showing micro-morphology of TMZ loaded PLGA-PLA wafers at different drug loading (10-50 wt%). E) Changes in the drug release pattern with increase in drug loading from 20 to 30 wt%.

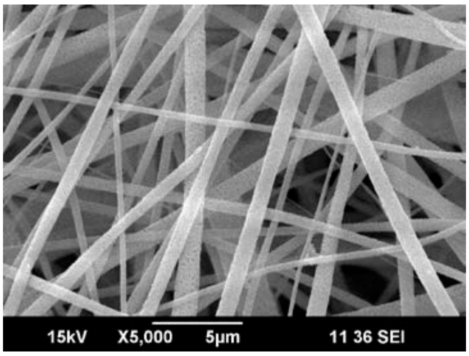

Figure S3. SEM image of nCP:Fe loaded theranostic wafer showing smooth micro-morphology without any bead formation.

Table S2. Haematological parameters of TMZ-SF wafer implanted animals.

	Normal Range	Untreated Control	TMZ-SR wafer implanted animals	
			1 Month	3 Months
RBC ($10^6/mm^3$)	6.67 – 9.75	9.3 ± 0.6	8.5 ± 1	6.7 ± 1
WBC ($10^3/mm^3$)	6.6 – 12.6	12.1 ± 3.5	10.5 ± 3.5	12.3 ± 0.1
Platelet (10^3/mL)	150 – 460	348 ± 53	140 ± 14	490 ± 57
Neutrophils ($10^3/mm^3$)	1.77 – 3.38	2.6 ± 0.6	3 ± 1.3	2.8 ± 0.1
Lymphocytes ($10^3/mm^3$)	4.78 – 9.12	9.9 ± 0.7	7.5 ± 1.3	9.5 ± 0.1

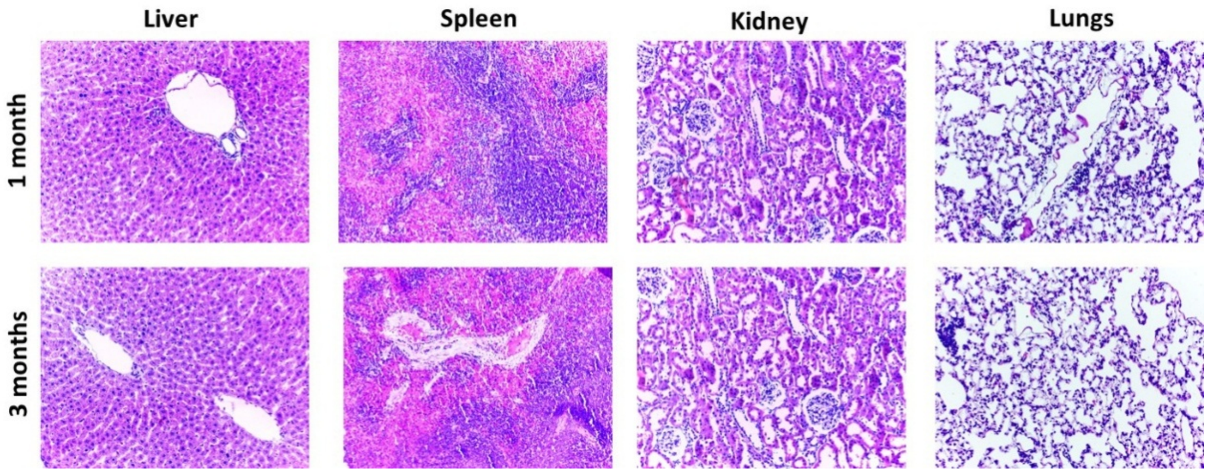

Figure S4. H&E stained sections of liver, spleen, kidney and lungs of rats implanted with TMZ-SR wafers at 1 and 3 months, showing no organ toxicity.

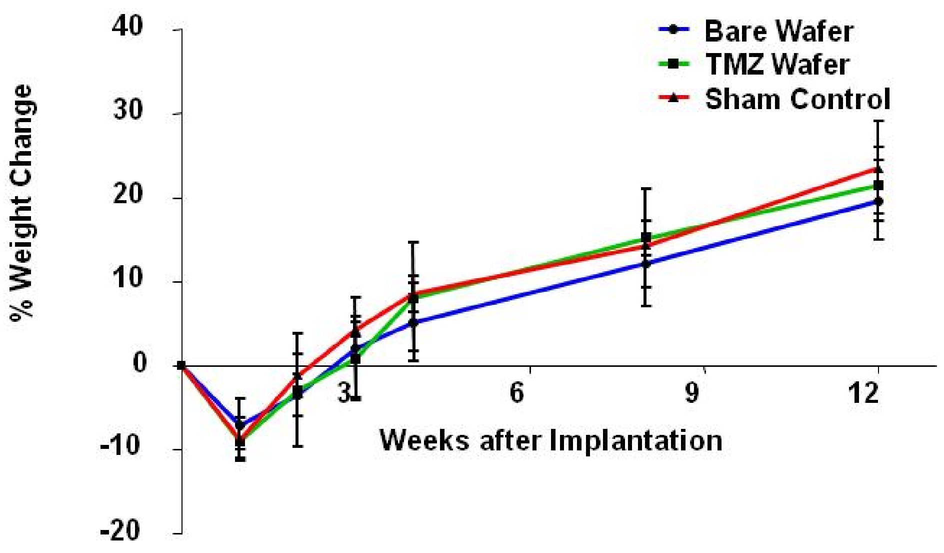

Figure S5. Graph showing body weight changes following implantation of nano-fiber wafer implant in brain cortex.

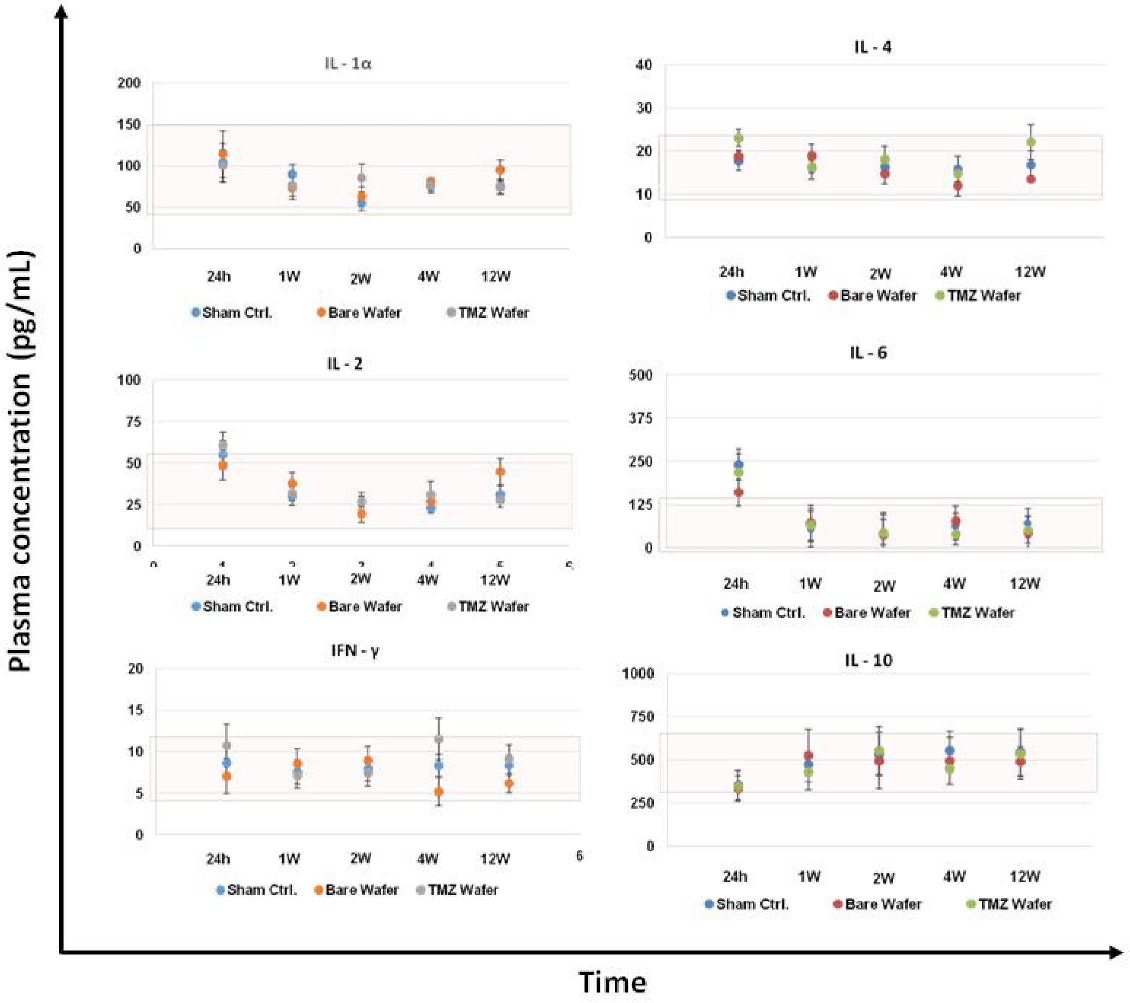

Figure S6. Plasma cytokine levels of sham, bare wafer and TMZ-SR wafer implanted animals at different time intervals (h = hour, W = week).

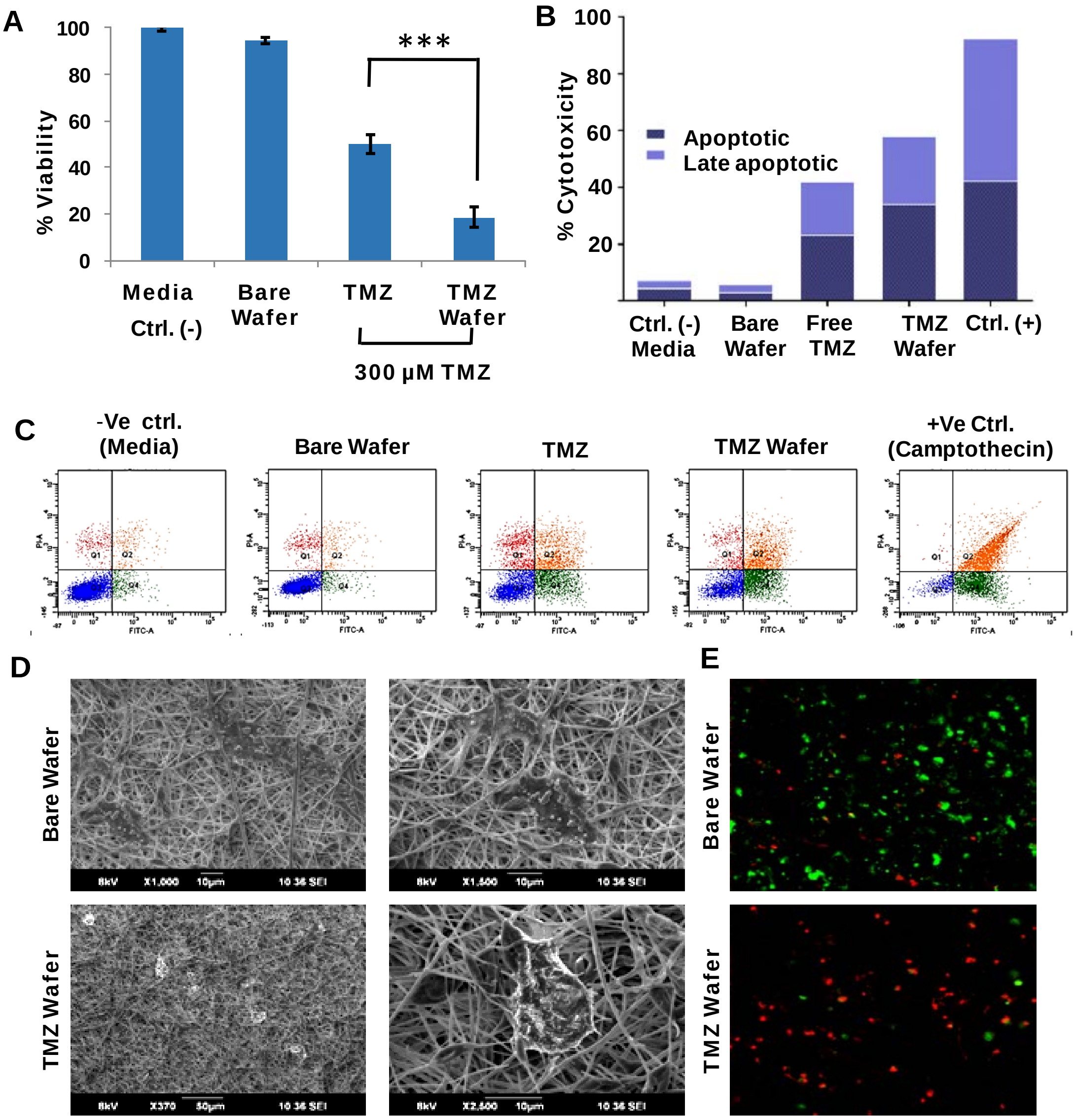

Figure S7. ***In vitro*** **cytotoxicity evaluation of TMZ wafers in U87MG human glioma.** (A) Graph showing enhanced cytotoxicity (81.5%) induced by TMZ-FR wafer compared to free TMZ (50.5%) as determined by MTT assay after 96 h treatment. (B) Graph and (C) dot blots depicting the flowcytometry results showing enhanced cell death in TMZ-FR wafer treated glioma cells at 96 h. 57.7% cells underwent apoptosis when treated with TMZ-FR compared to 41.7% in free-TMZ (D) SEM images of glioma cells on bare wafers (no drug) and TMZ-FR wafer showing impaired cell growth in the later (E) Confocal images of live-dead assay showing live glioma cells (green) in bare wafer and dead cells (red) in the TMZ-FR wafer.

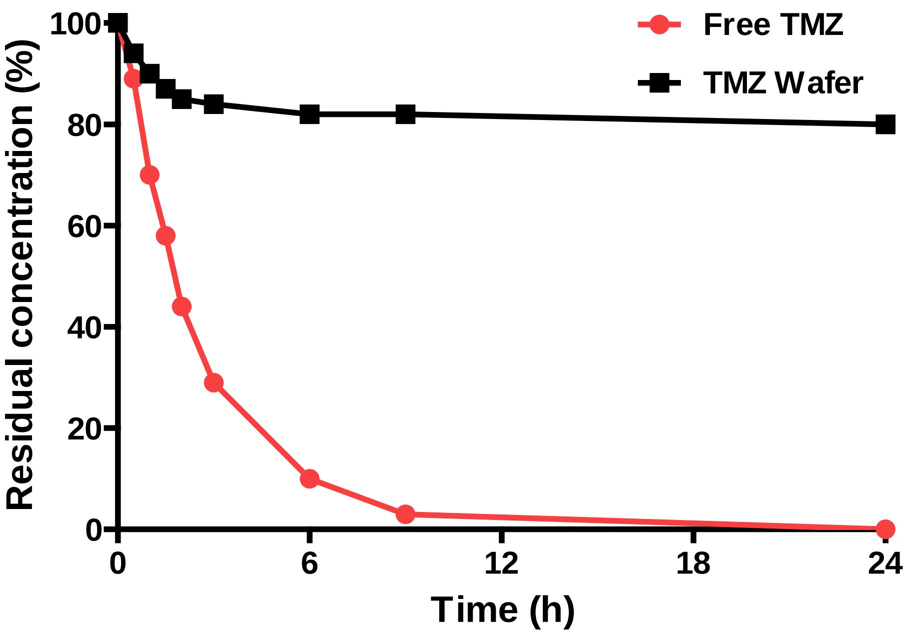

Figure S8. Degradation of free and wafer loaded TMZ in complete medium. Almost 100% free-TMZ was found decomposed by 9 h in serum whereas ~ 80% of TMZ remained intact in TMZ-FR implant, indicating the improved stability of TMZ by polymer encapsulation.

Video S1: Animal after three days of wafer implantation

Video S2: Animal after three months of wafer implantation
